# Satellite Network Task Deployment Method Based on SDN and ICN

**DOI:** 10.3390/s22145439

**Published:** 2022-07-21

**Authors:** Zhiguo Liu, Xiaoqi Dong, Lin Wang, Jianxin Feng, Chengsheng Pan, Yunqi Li

**Affiliations:** 1 Communication and Network Laboratory, Dalian University, Dalian 116622, China; dongxiaoqi3360@163.com (X.D.); fengjianxin863@163.com (J.F.); pcsdldx@163.com (C.P.); liyunqi_lyq@163.com (Y.L.); 2 School of Environmental and Chemical Engineering, Dalian University, Dalian 116622, China; further2002@163.com; 3 School of Electronics and Information Engineering, Nanjing University of Information Science & Technology, Nanjing 211800, China

**Keywords:** software-defined network (SDN), information-centric network (ICN), satellite networks, edge computing, task deployment

## Abstract

With the rapid development of 5G and the Internet of Things, satellite networks are emerging as an indispensable part of realizing wide-area coverage. The growth of the constellation of low-orbit satellites makes it possible to deploy edge computing services in satellite networks. This is, however, challenging due to the topological dynamics and limited resources of satellite networks. To improve the performance of edge computing in a satellite network, we propose a satellite network task deployment method based on SDN (software-defined network) and ICN (information-centric network). In this method, based on the full analysis of satellite network resources, a mission deployment model of a low-orbit satellite network is established. The genetic algorithm is then used to solve the proposed method. Experiments confirm that this method can effectively reduce the response delay of the tasks and the network traffic caused by task processing.

## 1. Introduction

The popularization and application of 5G have enabled the Internet of Things for terrestrial networks. In the 6G era, the “Internet of Everything” (IOE) will be realized. In terms of communication coverage, 6G communication scenarios are going to be extended to sea, land, air, and even underwater space. With the rapid development of voice recognition, intelligent transportation, the Internet of Things, and other applications, providing users with global anytime and anywhere network access is essential. Such access enables reliable service support. However, there are significant challenges to network coverage for the following scenarios: (1) oceans, deserts, and other areas with no Internet access capacity account for most of the Earth’s surface; (2) ground network equipment may be affected by natural disasters. At the same time, the base stations of mobile communication are all deployed in densely populated areas. Therefore, it is impossible to access the network through the existing ground mobile communication network in other places [1].

Satellite Internet is the third generation of Internet infrastructure following the wired and wireless Internet. Satellite Internet can achieve worldwide area coverage. It is almost unaffected by weather and geographical conditions and can work all day long. The analysis of Starlink by the Institute of Software of the Chinese Academy of Sciences concluded that the average end-to-end round-trip RTT of the low-orbit satellite network is 38.8 ms, which is comparable to that of the terrestrial fiber network. The adoption of low-orbit satellites can significantly reduce the communication delay to meet the needs of low-delay services. This also contributes to the miniaturization of ground equipment and the reduction of communication energy consumption of ground IoT equipment [2].

Although the satellite–ground collaborative network has the characteristics of wide coverage and strong disaster resistance, the traditional network architectures become unsuitable in the face of more complex traffic patterns and more flexible business needs. In addition, the transmission protocol in the traditional IP network makes it difficult to cope with the unstable communication quality and different service types in the satellite network scenario. To address this issue, researchers adopt the idea of the software-defined network (SDN), which reduces the network to a general software that is easy to maintain and modify and also more intelligent and efficient [3]. The SDN is a programmable and open network architecture that can speed up the development and deployment of network services, improve the intelligence and automation level of the network, simplify the network control protocol, and reduce the cost of maintenance and expansion. Using SDNs for the satellite networks provides a solution to address the increasingly complex business needs where an SDN controller is deployed to obtain the topology of the whole network [4]. The SDN technology simplifies the configuration and update of the underlying infrastructure and coordinates the operation, load, and data delivery across domains [5]. The information-centric network (ICN) is a subversive change to the traditional network [6]. The ICN is committed to replacing the “IP” layer with “content” [7]. Nodes in the ICN network cache the data passed during transmission according to the cache replacement strategy, so that the satellite network can significantly improve the response delay of the satellite network [8]. Furthermore, with the rapid development of onboard computing, intersatellite link, and other technologies, joint application of SDNs and ICNs to satellite networks is proposed. Specifically, the SDN technology reconstructs the architecture of satellite networks to better support the deployment of services and the management of satellite constellation topology [9]. The ICN technology also provides an efficient scheme for data transmission in the Internet of Things.

This paper proposes a satellite network task deployment method based on SDNs/ICNs to improve the user experience of satellite network access equipment. The ICN cache characteristics enable the cached communication data in the satellite network, which can be used for computing services. The SDN technology is also used to control the LEO satellite network and obtain the status of the available resources to coordinate the resource status of the network for the deployment of computing tasks. Taking the task delay and satellite traffic as the optimization objectives, a genetic algorithm is used to coordinate the status of network resources to deploy computing tasks.

## 2. Related Work

### 2.1. SDN and ICN Research on the Satellite Network

As an emerging network management mode, the SDN can effectively improve the resource management level and the overall performance of the satellite network. The SDN separates the control plane from the forwarding plane, which can simplify the design and configuration of the satellite. The researchers introduced the idea of SDN into the satellite network to improve the efficiency of network management and configuration and designed a multilayer network structure based on the SDN integrated with the ground [10]. This includes the multilayer network structure and the deployment of virtualization services. To improve the management performance of the SDN in a satellite network, researchers have considered the dynamic configuration of the SDN controller to ensure the optimal controller position [11]. The SDN can obtain the global view of the entire satellite network and then combine it with the NVF technology to realize the effective arrangement and configuration of network resources. In [12], combining SDN and NVF technologies, an on-demand satellite network slicing framework was proposed to realize a flexible configuration of resources and various services.

A satellite network is the supplement and extension of the ground communication network. Researchers try to integrate the ground network and satellite network to provide low latency and high-rate data delivery. For instance, in [4], the ground and satellite networks were integrated and the SDN technology was adopted to centralize and flexibly allocate spectrum resources. This confirms that the method based on the SDN provides obvious advantages in improving network throughput and reducing communication delay.

The ICN also decouples content and communication addresses through a location-independent communication mode. The application of ICN technology to satellite networks effectively eliminates the impact of satellite dynamics on communication links. Especially for the collection of extensive Internet of Things data, the traditional IP address-based communication mode is inefficient. Researchers use both SDN and ICN technologies in the satellite networks to add the characteristics of the cache in the ICN network to the satellite network. This improves the delivery delay of the content [13]. To cope with the rapid growth of satellite–ground network requests, researchers have designed an ICN-based architecture. By adopting cooperative caching and coding caching methods, satellite network traffic is reduced, and content retrieval efficiency is improved [14].

### 2.2. Satellite Networks Converging Edge Computing

Satellite networks are important supplements to achieve wide-area coverage. With the increasing demand for computing-intensive and Internet of Things applications, the idea of introducing edge computing into satellite networks becomes viable. Deploying edge computing services on the satellite networks extends computing services to the edge of the network. This provides nearby computing services for the satellite network access users and further improves the response speed of computing requests. With the satellite network communication mode based on the ICN, the data required for the calculation can also be obtained directly from the satellite network. In [15], possible methods of deploying edge computing on STN networks are studied and they design a task-parallel computing method to improve the QoS of the network. Limited energy consumption is also a prominent problem for satellite networks. Therefore, to provide low-latency computing services, one needs to incorporate the overall energy consumption of the satellite network. In the satellite IoT scenario, researchers try to deploy edge computing services on satellite networks. The time delay shows that edge computing and deep learning technology can increase detection delay and save backhaul bandwidth when processing IoT image data. Furthermore, by deploying edge services, multiple network resources such as computing, caching, and bandwidth in the network need to be reconfigured. Aiming at the problem of low management efficiency of multiple resources, ref. [16] proposed a dynamic resource allocation framework for fine-grained resource management.

The introduction of edge computing services into satellite networks also brings new key technical issues. Task deployment is a technology that plays an essential role in completing computing services. There are many nodes in the satellite constellation and the resource status of the node is uneven. However, the completion of computing tasks requires comprehensive consideration of the consumption of multiple resources. The LEO satellite constellation is widely used for the coordinated deployment of multitasks due to its large number and computational load. Compared with the ground network, the task deployment in the satellite network needs to consider the topological dynamics of the satellite network and the limited resources. For instance, ref. [17] approached this issue from the perspective of overall load balancing and proposed a heuristic algorithm to find the optimal task allocation plan. In addition, ref. [18] combined the joint resource allocation problem of space and ground to design a task allocation plan and incorporated the dynamics of the system to design an algorithm based on reinforcement learning to learn the best allocation strategy.

To sum up, the research on SDN and ICN satellite networks and edge computing task deployment has been relatively mature. However, there are few studies on the combination of edge computing technology and SDN/ICN satellite networks and there are few studies on the relevant deployment algorithms. In the future, SDN/ICN satellite networks will be called mainstream networks, so it is necessary to study the SDN and ICN satellite network task deployment methods.

## 3. System Model and Problem Formulation

### 3.1. Scene Overview

With the operation of the 5G mobile communication network, the use of IoT devices based on mobile communication systems will also grow rapidly [19]. The base stations of the ground mobile network are often deployed in densely populated areas. In areas without ground network coverage, there are also access requirements for IoT equipment, such as forest, ocean, air, and other scenes, so a satellite communication system is used to collect IoT data in this area. Compared with a geostationary orbit (GEO) satellite, a low-orbit (LEO) satellite can reduce the propagation delay and transmission loss. This is helpful for saving the energy consumption of IoT equipment in remote areas. In addition, the global seamless coverage can be achieved through the constellation composed of multiple LEO satellites. This improves the coverage of the satellite network and alleviates the difficulty of GEO satellite orbit position and frequency coordination.

**Figure 1:**
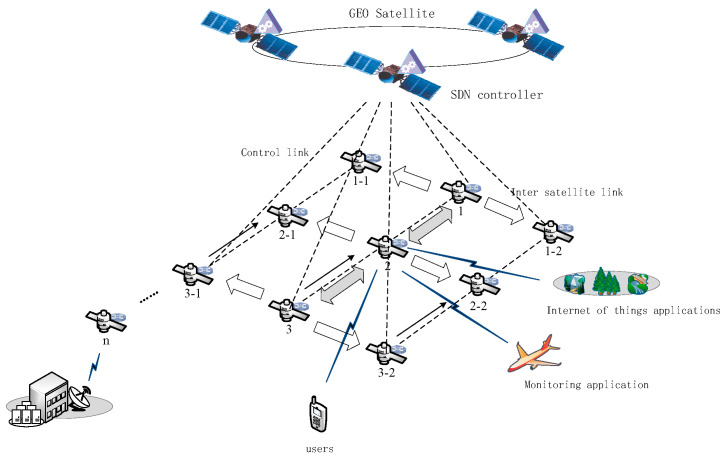
The satellite network scenario.

**Figure 2:**
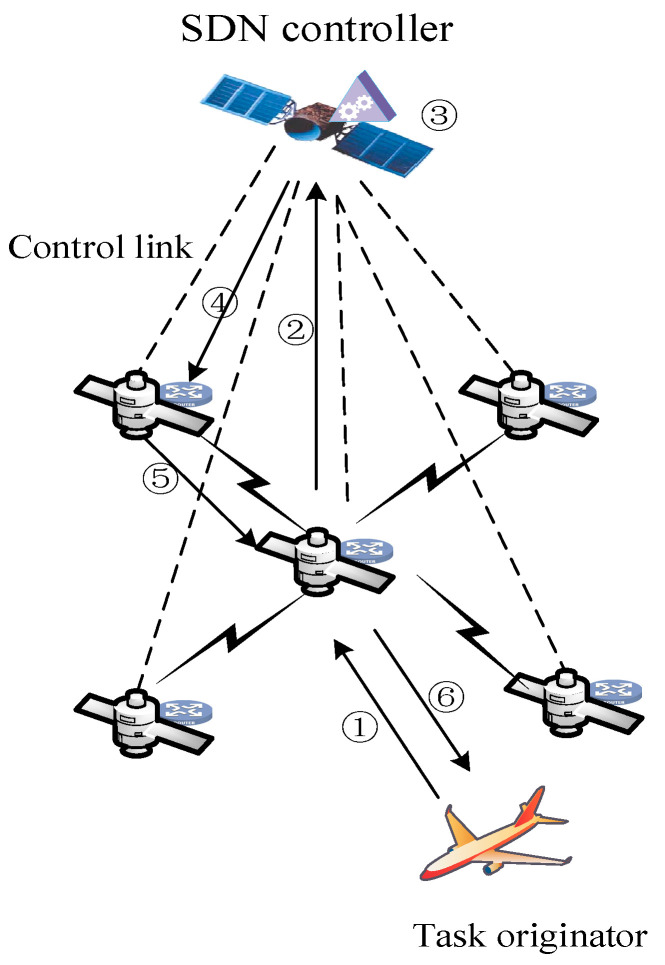
The SDN-based task distribution diagram.

To enable high-efficiency control of the low-orbit satellites, a layered satellite networking scheme is considered. GEO satellites are widely used in the control layer of hierarchical satellite communication systems because of their wide area coverage and high load capacity. In this approach, the GEO satellite is mainly responsible for the topology monitoring and flow monitoring of the LEO constellation. Compared with transmitting all the original data to the ground station for processing, the onboard calculation can significantly reduce the communication volume of intersatellite links and reduce the processing delay of the tasks. Two kinds of application scenarios of satellite network are considered, including providing traffic services and computing services for users and the satellite Internet of things—wherein the ground IoT equipment uploads the data to the low-orbit satellite node and then transmits the processed data to the ground station after in-orbit integration. Figure 1 is a schematic diagram of the satellite network scenarios. The SDN controller is deployed to GEO Satellite and the network scenario includes user terminals, data monitoring devices, Internet of things devices, and other devices to access the LEO satellite network. The LEO satellite undertakes the tasks of data forwarding, content caching, and computing services of the access devices. It is assumed that each LEO satellite has only four communication links: the communication links with adjacent satellites in the same orbit and the communication links between satellites in different orbits. The local processing performance of the access devices cannot meet the computing requirements. Therefore, this needs to be transferred to the LEO satellite network for processing. The task processing flow based on SDN and ICN Technology is presented in Figure 2.

In the communication mode based on the ICN, the communication between end-to-end devices is based on two kinds of transmission packets: interest packets and data packets. Based on the global naming mechanism, the content has a globally unique name identifier. After receiving the packets, the nodes in the communication link cache them in the current node according to a certain strategy. The onboard cache greatly improves the response time when the content is requested again.

In this paper, the idea of edge computing is introduced into the satellite network and the problem of computing offload in satellite networks is considered. As shown in Figure 2, step one is for the task initiator to distribute the task to the connected LEO satellite. Step two is to send mission requirements to the GEO satellite, namely the SDN controller, for the mission receiving satellite. The SDN controller collects the resources and link states of the LEO nodes. In step three, the SDN controller decomposes the task into smaller subtasks and performs the task deployment method proposed in this paper to find the optimal placement position of the subtasks. In step four, the SDN controller sends the subtasks to the task deployment satellite nodes through the control link. The computing data required by the task are decided by the SDN. The path between the content source node and the destination is then constructed by distributing the flow table to complete the data search process. Step five means that, after the task is calculated at each task deployment point, due to the dynamic nature of the satellite and the task initiator, there may be a great time delay from task start to task completion. Therefore, it may cause the task initiator to change its position and connect to different LEO satellite edge nodes when the task is completed. Therefore, here, it is necessary to determine the coverage satellite node of the task initiator through the SDN controller on the GEO with a wider coverage, and then send it back to the satellite by each task deployment point. Then, the satellite node integrates the subtask results. Step six is to return the task result to the task initiator.

A graph structure used G(V,E) to represent the network topology of the LEO satellite network when the task is initiated. In this setting, V(G) represents the set of LEO satellite nodes within the allowable range of network delay, and E(G) denotes the intersatellite link of the satellite nodes. Furthermore, $M=\left\{m_{1},m_{2}\ldots m_{n}\right\}$ represents the set of all satellite numbers of the whole network and $L_{m}(e_{m},v_{m})$ denotes the resource status that the LEO satellite node can provide. We also define $e_{m}$ as the computing resources that the LEO satellite node can provide, and *v_m_* as the cache space that the LEO satellite node can provide.

In addition to the edge computing nodes and cache nodes on the satellite, the ground cloud computing center should also be considered in this paper. The ground cloud computing center is centralized processing and the edge computing can be edge processing, complementing each other. Because the computing power of the edge computing node is very limited, if the satellite edge computing resources are insufficient, the task can be sent to the remote cloud for processing. The remote cloud computing center has rich storage and computing resources. The cloud computing center and satellite edge computing nodes cooperate with each other to achieve center–edge collaboration, networkwide computing power scheduling, networkwide unified management and control, and truly provide “ubiquitous” services. Therefore, this paper sets that the computing resources and cache resources provided by it exceed the sum of the resources of the whole LEO satellite network.

In this paper, computing tasks are divided into computing intensive and resource intensive according to requirements. The analysis of monitoring data for tasks that consume large amounts of computing resources, such as unprocessable computing tasks, is classified as computing intensive. The tasks of video compression processing and big data analysis in the IoT scene are classified as resource intensive. Computing intensive applications consume a lot of computing resources of satellite edge computing nodes, while resource intensive applications occupy a lot of on-board storage resources and intersatellite link bandwidth brought by data transfer. Therefore, the optimization goal of this paper is to minimize the task processing delay and the traffic between intersatellite links.

### 3.2. Task Model

#### 3.2.1. Computing Task Decomposition

Due to the limited computing resources, a single satellite is unable to independently complete the whole task. Therefore, we consider decomposing the computing task and deploying the decomposed subtasks to multiple LEO satellite nodes for collaborative processing.

Task decomposition is represented by subtask tree and subtask association. The relationship of subtasks is represented by a directed single-in-single-out acyclic graph (DAG). The entry of the DAG graph represents the initial input of the program, and the input data of each subtask are searched independently after the task of each subtask is generated. The exit of the graph represents the output of the whole program. There are parallel and serial relationships between the subtasks. If the relationship between subtasks is parallel, the subtasks execute the corresponding tasks independently. If it is a serial relationship, the execution of subtasks can only be performed after the precursor task is completed.

The whole task is divided into different layers. Each layer task is divided into several parallel subtasks according to the parallel relationship. After the partition, the resource consumption of a single subroutine is evaluated. If the resource consumption exceeds the system load, the parallel analysis will lead to a large number of parallel tasks in each layer, which reduces the response time of tasks. If the program cannot decompose the parallel relation, it is decomposed into serial subroutines according to the amount of computation. After task decomposition, the task initiation node $O=\{task_{1},task_{2},\ldots ,task_{n},G\}$ is used to represent the decomposed subtasks and the relationship between subtasks.

#### 3.2.2. Task Model

After the task is decomposed into several subtasks, the resource consumption of each subtask is represented by $task_{i}(C_{i},R_{i},G)$. We define $C_{i}$ as the amount of computation required by subtask i, and $R_{i}$ is the cache space required by the task i. The computing resources required by the task include the following: (1) The data of monitoring devices accessed in the IoT scenario need to be obtained from the access devices when performing the calculation; (2) In the case of providing computing services for access device terminals, part of the data required for computing comes from the access device, and the other part comes from the ground data center.

### 3.3. Communication Model

To balance the communication load caused by onboard computing, it is necessary to model the network traffic. It is assumed that all network devices are connected to the task initiator through the satellite–ground link. In the satellite IoT scenario, a large number of monitoring data cannot be stored in one node, yet this can be stored in multiple nodes in the network. Considering that the satellite network provides computing services for the terminal, the computing resources need to be obtained from the terminal and the ground cloud computing center. Therefore, the ground cloud computing center is treated as a node equivalent to a satellite.

The data required by subtask $task_{i}(C_{i},R_{i},G)$ need to be obtained from satellite nodes j. The amount of resources that a satellite can provide is $cache_{1}$, $cache_{2}$…$cache_{j}$. The node providing resources to the deployment point of the task needs to go through $k_{1}$, $k_{2}$$\cdots k_{j}$ times. Therefore, the communication volume $F_{i}$ for a single subtask transmission is:

(1) $$F_{i}=\sum_{w=1}^{j}k_{w}cache_{w}=k_{1}cache_{1}+k_{2}cache_{2}+\ldots +k_{j}cache_{j}$$

The amount of communication caused by completing the task resource transmission composed of n subtasks is as follows:

(2) $$F_{n}=\sum_{i=1}^{n}F_{i}$$

In the communication mode based on IP addresses, the network relies on the address to transmit messages. Network nodes cannot perceive content. This makes it infeasible for the satellite nodes to acquire the data content required by the mission. The content name-based transmission mode of the ICN architecture enables nodes to have the ability to sense the local cache content. The cache content is also placed in the satellite network to reduce the time required to obtain the computing data required by the task and the task processing delay.

In the SDN-based satellite network architecture, the SDN controllers are deployed on the GEO satellites. The LEO satellite node can sense the local cache content and upload the cache update to the SDN node in time. The data needed by subtasks are searched globally through the SDN. The node sends the find message to the GEO satellite node to find the required data. The SDN controller obtains the dynamic link between LEO satellites in real-time and returns the data to the node requesting the data.

### 3.4. Task Model

#### 3.4.1. Delay in Task Deployment

The distance between LEO satellites is constantly changing. Therefore, the communication between satellites needs to consider the influence of distance. The link length D between the satellites can be calculated from the positions between satellites and presents periodic changes when the constellation is fixed. Assume that D(t) is the function of intersatellite distance changing with time, and the communication delay can be expressed as:

(3) $$t_{d}=\frac{D(t)}{c}$$

where D(t) is a function which can be generated by the constellation operation law. In (3), c is the transmission speed of the electromagnetic wave in a vacuum.

The delay of a single transmission is $t_{d1}$, $t_{d2}$…$t_{dn}$, respectively. Therefore, the delay of a single subtask deployment is:

(4) $$t_{b}=t_{d1}+t_{d2}+\ldots +t_{dn}$$

Tasks are composed of subtasks, and the deployment delay of a single subtask is $t_{b1}$, $t_{b2}$…$t_{bn}$, and the deployment delay of task $Z_{n}^{1}$ is:

(5) $$Z_{n}^{1}=\max\left\{t_{b1},t_{b2}\ldots t_{bn}\right\}$$

#### 3.4.2. Delay in Task Data Transmission

The transmission of the data required by the task to the task deployment node needs to be transmitted by multiple satellites. The communication bandwidth between any two satellites is, however, different. Therefore, they need to be calculated separately. Let $M=\left\{m_{1},m_{2}\ldots m_{n}\right\}$ be the collection of all satellite numbers in the network. If there is a link between the satellite nodes numbered m and n, the transmission rate between the satellite nodes numbered m and n is $r_{m}^{n}$. Therefore, the delay to complete the transmission of a data volume of $W^{m}$ is:

(6) $$T_{m}^{n}=\frac{W^{m}}{r_{m}^{n}},\;\forall m,n\in M$$

Using the whole network of an SDN, the path that the task needs to take is then determined and the transmission delay between any two satellites can be calculated by (6). Assume that the satellite number is *m*, *n*, *b*… *x*; *h* is the route, the forwarding times are k, and the communication delay is:

(7) $$Z_{k}=T_{m}^{n}+T_{n}^{b}+\ldots +T_{x}^{h}$$

Because the transmission is synchronous, the transmission delay is the longest subtask transmission time. Assume that the forwarding times of a single subtask are $O_{1}$, $O_{2}$…$O_{n}$, then the transmission delay is:

(8) $$Z_{n}^{2}=\max\left\{T_{o_{1}},T_{o_{2}}\ldots T_{o_{n}}\right\}$$

#### 3.4.3. Task Computation Delay

Because each satellite node can initiate task calculation, multiple subtasks are inevitably deployed in one node. The satellite node evaluates the computation latency of the task by obtaining the local computing task queue. The resources of a single LEO satellite are, however, very limited. Therefore, it is necessary to consider the computing and caching resources that a single satellite can provide when deploying a mission. Here we assume that the onboard processor is a single-core, single-threaded processor. Each satellite has a task queue, and the tasks in the queue have three states: running, ready, and waiting. The ready state indicates that the task has completed collecting the data required by the task and is waiting to obtain the CPU usage. The waiting state indicates that the computing task has been deployed to the node, but the data required by the task has not been collected, or the precursor task has not been completed, waiting for the output and completion signal of the precursor task. When the task completes data collection, it switches to the ready state. If $F_{m}$ is the clock frequency of the LEO satellite and $f_{m}$ is the clock frequency assigned to the subtask, the delay required to complete the subtask $task_{i}(C_{i},R_{i},G)$ is:

(9) $$t_{l}=\frac{R_{i}}{f_{m}},f_{m}\leq F_{m}$$

The data capacity required by the subtask should also be less than the capacity that the satellite node can provide.

(10) $$R_{n}\leq v_{m},\forall m\in M$$

The task computation delay includes the waiting delay in the queue and the computation-required delay. The waiting time is the time for the satellite node to complete the task of the current calculation queue. The waiting delay $t_{w}$ is calculated by the satellite node and uploaded to the SDN control node. Therefore, the time delay is:

(11) $$Z_{n}^{3}=t_{w}+t_{l}$$

The delay of task processing includes the delay of task deployment, the delay of data transmission, and the delay of task computation. The total deployment delay of the task is:

(12) $$Z_{n}=Z_{n}^{1}+Z_{n}^{2}+Z_{n}^{3}=\max\left\{t_{b1},t_{b2}\ldots t_{bn}\right\}+\max\left\{T_{o_{1}},T_{o_{2}}\ldots T_{o_{n}}\right\}+t_{w}+t_{l}$$

Deploying edge computing on the LEO satellite network improves the response time of terminal computing tasks and reduces the amount of traffic back to the ground-based cloud computing center. However, the communication burden of the LEO satellite network is increased due to the mission deployment and the data transmission required by the mission. Note that task processing needs to also consider QoE. Therefore, this paper combines the communication volume and task response delay caused by edge calculation to develop the joint objective function:

(13) $$Q_{n}=\alpha Z_{n}+\beta F_{n}$$

where α,β∈[0,1] and α+β=1. α and β are the balanced relationship between task processing delay and task traffic. For delay-sensitive tasks with large computation and small communication volume, α and β should be increased and decreased, respectively. For tasks with large communication volume, such as video service, which are less sensitive to time delay, β should be increased and α decreased. Therefore, these parameters should be set dynamically during task deployment according to task requirements.

According to the above modeling, SDN-based satellite network task deployment involves resource allocation amongst multiple satellites, and there are many ways to deploy a single task. If the number of task deployment nodes is M and the number of tasks is N, the time complexity is $O(M^{N})$; hence, it is a complex combinatorial optimization problem. Therefore, the optimization goal in this paper is an NP-complete problem—that is, the time complexity in the worst case increases exponentially with the scale of the problem. Therefore, the task deployment strategy cannot find the optimal solution in polynomial time complexity. Exhaustive search methods and branch and bound methods can solve this problem, but their complexity is prohibitive. Therefore, a heuristic algorithm is considered to achieve an approximate solution in the allowable time. A genetic algorithm is commonly used in solving complex combinatorial optimization problems. Compared with traditional optimization methods (enumeration, heuristic, etc.), a genetic algorithm is simple and easy to implement. It takes biological evolution as the prototype, and the time complexity is related to the number of iterations and the number of populations. The time complexity of the genetic algorithm is $O(n^{2})$, which demonstrates quick convergence and a high degree of robustness. For a given required accuracy, a genetic algorithm can find the approximate optimal solution.

## 4. Task Deployment Method Based on Genetic Algorithm

**Figure 3:**
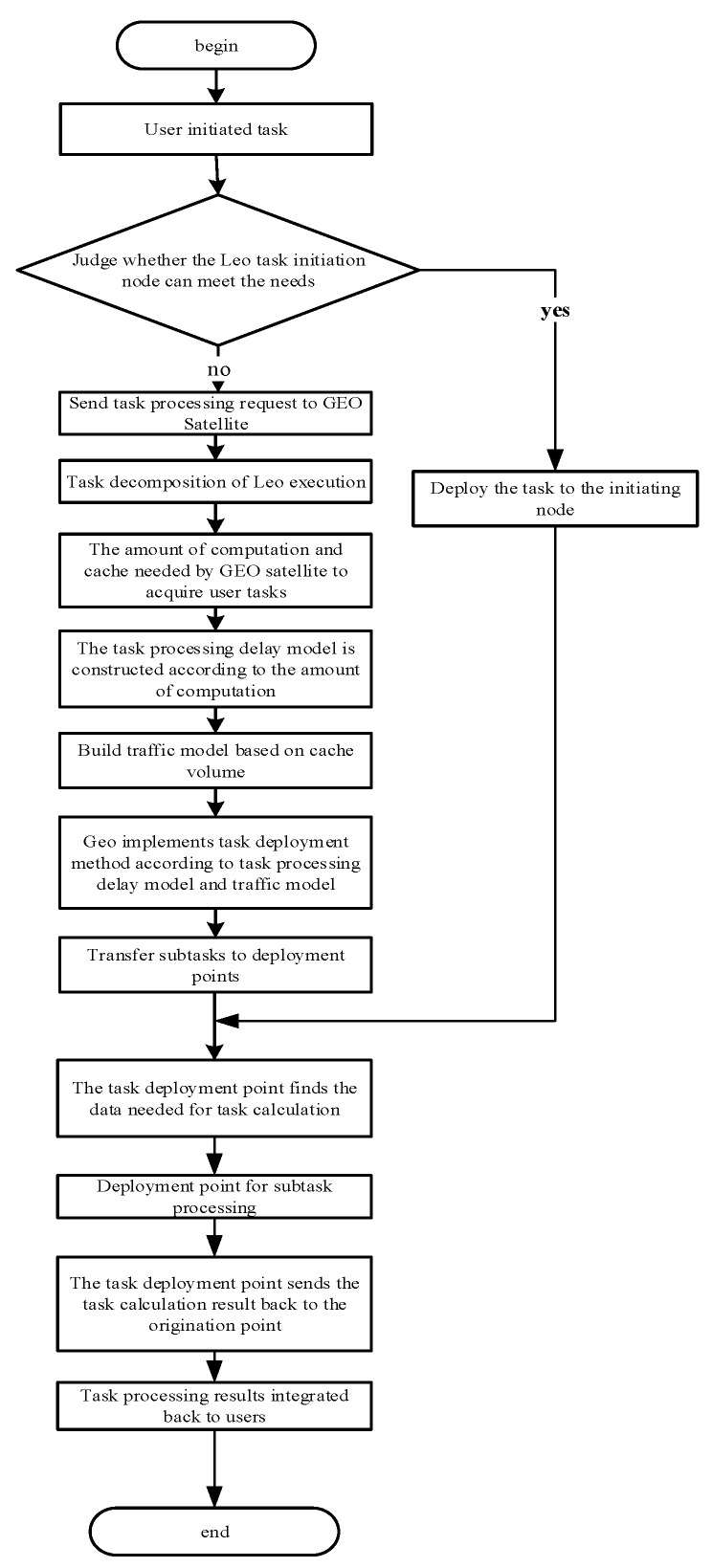
The flowchart of the task deployment method.

The complete process of task deployment is shown in Figure 3.

Task deployment of a satellite network based on the SDN involves resource allocation among multiple satellites. The deployment path of a single task has a variety of ways, so the task deployment strategy cannot find the optimal solution within the polynomial–time complexity. Therefore, a heuristic algorithm is considered to achieve an approximate solution within the allowable time. A genetic algorithm imitates the principle of biological evolution and has advantages in solving search problems. The steps of a task deployment strategy include coding, fitness calculation, selection, crossover, and mutation.

The satellite deployment strategy mentioned above refers to the problem of transferring user-initiated computing tasks to multiple satellite nodes. This is according to the path under the centralized decision of the SDN controller under the condition of limited resources of a single satellite. The task deployment strategy mentioned in this paper needs to meet the following prerequisites: 1. The design of satellite nodes needs to be based on the idea of the ICN, such that the nodes can perceive the content of local resources; 2. under the SDN satellite network architecture, the LEO satellite can quantify its resources for calculation, caching, and communication, and can upload the available resource information to the SDN controller in real-time.

### 4.1. Coding

The number of chromosomes in the initial population is set as $N^{c}$, and the set is used to represent the population. An m×n task deployment matrix is used to represent an individual as a feasible solution to the problem. Let *M* be the number of satellites that can provide resources for calculation and caching when making decisions, and N is the number of subtasks that the current task is decomposed into. An individual in a population is represented as:

(14) $$A=\left[\begin{array}{cccc} a_{1m_{a}} & a_{1m_{b}} & \cdots & a_{1m_{m}}\\ a_{2m_{a}} & a_{2m_{b}} & \cdots & a_{2m_{m}}\\ \vdots & \vdots & \ddots & \vdots \\ a_{nm_{a}} & a_{nm_{b}} & \cdots & a_{nm_{m}} \end{array}\right]$$

Each element in the individual matrix has a binary value, $a_{mm_{a}}=1$, indicating that subtasks with task numbers m are deployed on the satellite node with task numbers $m_{a}$. The computational and storage requirements of each subtask are different. Therefore, after the random generation of individuals (feasible solutions), it is necessary to detect whether the deployed nodes can meet the requirements of the computational amount and storage amount of the task. The individuals that cannot meet the requirements are then eliminated. Set is used to represent the population, and the number of individuals in the initial population is set as $N^{c}$. $N^{c}$ is a feasible solution set composed of the individuals.

### 4.2. Choose

The goal of a task deployment policy is to reduce the task response function and the traffic in the network. This is performed by adjusting the weight of the balance coefficients α,β according to the type of task. The fitness of individuals in each generation population is calculated by taking the utility function as the fitness standard of the individuals. The fitness function of the individuals is:

(15) $$Fit(A)=\frac{1}{Q_{n}}=\frac{1}{\alpha Z_{n}+\beta F_{n}}$$

A roulette strategy is used to represent the probability of individual selection. Based on this strategy, the higher the fitness value, the higher the probability of individual selection. The probability of individual selection is

(16) $$P(A)=\frac{Fit_{j}(A)}{\sum_{i=1}^{N_{c}}Fit_{i}(A)},\;\forall j\in N_{c}$$

### 4.3. Cross

Because individuals are represented by the task deployment matrix, interchanging the matrix rows is used to represent individual crossover operations. Each row of the task deployment matrix represents the deployment location of a single subtask, and the matrix row interchange can produce two new individuals. Two individuals are randomly selected in the population to swap rows and the probability of individual crossover is *P_c_*. The operation process is as the following.

The initial individual is expressed as:

(17) $$A_{g}^{1}={\left[\begin{array}{cccc} a_{{1m}_{a}}^{1} & a_{{1m}_{b}}^{1} & \cdots & a_{{1m}_{m}}^{1}\\ a_{{2m}_{a}}^{1} & a_{{2m}_{b}}^{1} & \cdots & a_{{2m}_{b}}^{1}\\ \vdots & \vdots & \ddots & \vdots \\ a_{{nm}_{a}}^{1} & a_{nm_{a}}^{1} & \cdots & a_{nm_{a}}^{1} \end{array}\right]}_{,}$$

The individual after crossover is represented as:

(18) $$A_{g}^{1}={\left[\begin{array}{cccc} a_{{1m}_{a}}^{1} & a_{{1m}_{b}}^{1} & \cdots & a_{{1m}_{a}}^{1}\\ a_{{2m}_{a}}^{2} & a_{{2m}_{b}}^{2} & \cdots & a_{{2m}_{b}}^{2}\\ \vdots & \vdots & \ddots & \vdots \\ a_{{nm}_{a}}^{1} & a_{{nm}_{a}}^{1} & \cdots & a_{{nm}_{a}}^{1} \end{array}\right]}_{,}A_{g}^{2}=\left[\begin{array}{cccc} a_{{1m}_{a}}^{2} & a_{{1m}_{b}}^{2} & \cdots & a_{{1m}_{m}}^{2}\\ a_{{2m}_{a}}^{1} & a_{{2m}_{b}}^{1} & \cdots & a_{{2m}_{b}}^{1}\\ \vdots & \vdots & \ddots & \vdots \\ a_{{nm}_{a}}^{2} & a_{{nm}_{a}}^{2} & \cdots & a_{nm_{a}}^{2} \end{array}\right]$$

### 4.4. Variation

Variation refers to the change of gene values at some loci of individual strings in a population. The genetic algorithm introduces mutation for two purposes: one is to make genetic algorithm have local random search ability. When the genetic algorithm has approached the neighborhood of the optimal solution through the crossover operator, the local random search ability of the mutation operator can accelerate the convergence to the optimal solution. According to the different representation methods of individual coding, there can be the following algorithms: (1) Real value mutation; (2) binary variation. Generally speaking, the basic steps of mutation operator operation are as follows: (1) Randomly select mutation bits for mutation; (2) judge whether the variant is a feasible solution. According to the form of the solution in this paper, binary mutation is adopted, and the probability of individual mutation is set as 0.01. 

Therefore, the satellite network task deployment method based on genetic algorithm is shown in Algorithm 1:
**Algorithm 1. Satellite Network Task Deployment Method Based on Genetic Algorithm****Input**: List of available satellite nodes downstream of satellite nodes *L* and network topology diagram between nodes $G_{L}$, network status diagram between available satellite nodes $G_{T}$, task decomposition diagram, list of available resources of nodes $L_{s}$, balance coefficients *α*, *β*.Initial population; Generate a matrix of m×n individuals and a population of $N^{c}$$C_{e}$.Parameters of the genetic algorithm: iteration number MaxGen, iteration state Gen=0; The crossover probability is $P_{c}$ and the mutation probability is $P_{v}$.**While** gen<MAXGEN **then**  Calculate the fitness Fit(A) of each individual, order the individuals according to fitness and copy the individuals with the highest fitness into the next generation population.  Select two individuals $A^{1}$ and $A^{2}$ with probability P(A) for crossover operation, and judge whether the individuals $A_{new}^{1}$ and $A_{new}^{2}$ generated after crossover meet resource constraints. Detect individual $A_{new}^{1}$,$A_{new}^{2}$:  **If** Individuals meet resource constraints **then**:       Insert $A_{new}^{1}$,$A_{new}^{1}$ into the population $C_{e}$;   **Else**       Re-perform the crossover operation;  **End If**  The mutation operation is carried out with the probability $P_{v}$, and the individual A generated after the mutation is $A_{v}$;  **If** $A_{v}$ meet resource requirements **then**:       Insert $A_{v}$ into population $C_{e}$;   **Else**       Delete $A_{v}$;  **End If**  Gen = gen + 1;  Preserve the fittest individuals of each generation.**End While**
**Outputs**: the individual with the highest fitness, the list of subtask deployment locations $L_{d}$, and the value of the minimum utility function $Q_{n}$.

## 5. The Simulation Result

### 5.1. Imulation Scenario and Parameter Design

**Figure 4:**
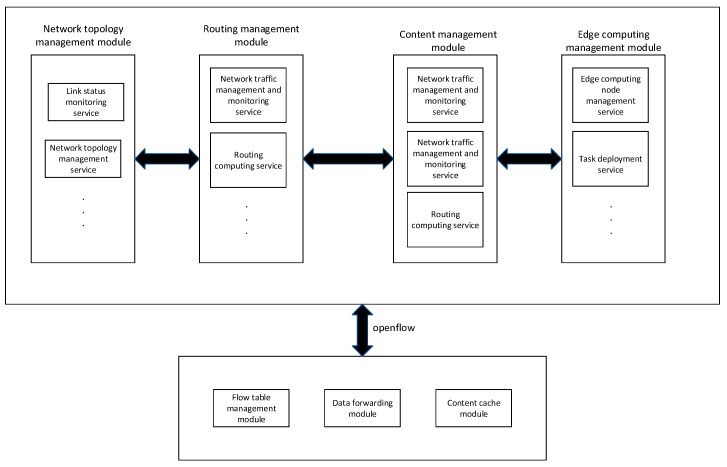
SDN/ICN architecture design.

Because the SDN architecture in this paper integrates ICN-related services, the application layer of the SDN can complete the related development of ICN services through software programming and then deploy it from the north interface to a GEO SDN controller. In Figure 4, the four main modules in the SDN controller in this simulation experiment are listed, respectively. The network topology management module and routing management module are the same as the traditional SDN controller, which are mainly responsible for topology-related and routing-related services. In addition, the SDN controller integrates the content management module, in which the content fragmentation management service is mainly used for fragmentation of large content, and then caching. Name resolution mainly establishes an index relationship between the content and the cache location, and obtains the location of the content through name resolution so as to distribute the content to the requester. The last is the content cache management service, which mainly monitors and manages the cache. Because the SDN distributes the flow table based on the openflow protocol switch to realize the functions related to routing, it only needs to add the content caching function to the flow table [19]. Finally, in order to realize the related functions of edge computing task deployment, the edge computing management module is added to the SDN controller, where edge computing node management service is responsible for the monitoring and management of edge computing nodes. When the task arrives, the task deployment module needs to obtain the global topology from the network topology management module, the cache of each satellite node from the content management module, and the node information from the edge computing node management module. Finally, the genetic algorithm proposed in this paper is used to solve the deployment location of the task.

To evaluate the performance of the proposed satellite network task deployment method, we carried out the following two simulation experiment parts. One part was to test the connectivity of the satellite network, and the other part was to test the performance of the mission deployment method.

**Figure 5:**
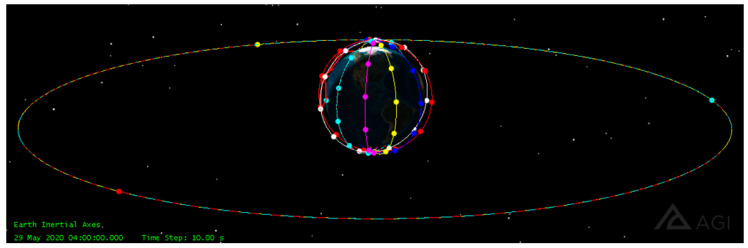
The 3D view of satellite constellations.

In the simulation experiment, the constellation composed of three GEO satellites can cover the whole constellation of LEO satellites. The SDN controller was deployed on the GEO satellite constellation to realize the global monitoring of the LEO satellite status. The SDN controllers of the three GEO satellites synchronized their data with each other and provided services for the LEO satellites covered by them. The 3D view of the satellite constellation is shown in Figure 5. The LEO satellite constellation adopted the Walker constellation with 66 satellites, which is divided into 6 orbital planes with an orbital inclination of 90 degrees, with 11 satellites distributed on each orbital plane. The edge computing server randomly selected two in each track.

In the ICN-based communication mode, the communication data can be cached on the satellite. In order to eliminate the impact of satellite network dynamics on the cache, the orbital inclination of the LEO satellite was set to 90 degrees, so that communication data could be cached on the same orbital surface with fixed intersatellite links. The satellite network deployment method proposed in this paper serves the equipment without ground network coverage. Moreover, satellite network edge computing cannot be supported without cloud computing centers on the ground. Therefore, we did not test the connectivity of the communication link between the ground network coverage equipment and the ground cloud computing.

Next, we will describe the satellite constellation in detail.The LEO satellite constellation was set to Walker 66/6/1, each satellite was at an altitude of 890 km, and the probe half-cone Angle of the sensors on the satellite was set to 45°. The sensor detection half-cone Angle of the three GEO satellites was set to 10°. We created a LEO intersatellite link to connect the ground cloud computing center to areas without ground network.

**Figure 6:**
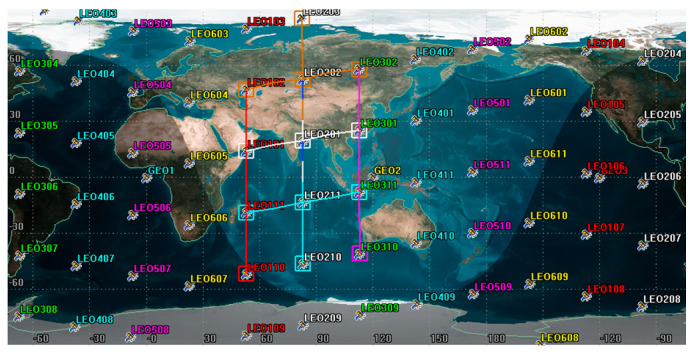
The 2D view of intersatellite link.

Mission deployment requires stable intersatellite link support. A single satellite is set to have four neighborhood communication links, two of which are used to establish fixed intersatellite links with front and back satellites on the same orbit. The other two links are used to establish unstable links with LEO satellites on the adjacent orbital plane. Figure 6 shows the 2D view of LEO intersatellite links after setting. We selected one of the satellites and tested the status of the satellite’s four communication links within 24 h, as shown in Figure 6.

### 5.2. Analysis of Task Deployment Method Based on Genetic Algorithm

Here, we simulated the task deployment method of a satellite network based on the SDN. The simulation parameters include task size, link state, node state, and so on. To describe the computing speed of the processor, the concept of MIPS (million instructions per second) was introduced into the simulation, which means the number of million instructions per second executed by the processor. The simulation parameters are set as shown in Table 1.

The processing speed of the LEO satellite’s processor was set to MIPS, and the processing speed of the ground computing center was set to MIPS. The available capacity of the onboard cache space was randomly distributed between 20 MB and 100 MB. The computation amount of the task was set to be between MI, and the input data amount of the task was set to be between 20 and 50 MB. Each task was decomposed into up to five dependent subtasks. The maximum bandwidth of the intersatellite link was set to 100 Mbps. The above-ground bandwidth and downlink bandwidth were set as 200 Mbps. In addition to providing edge services, satellite networks also need to maintain normal communication business. Therefore, the actual availability of resources was evenly distributed between 0% and 100%.

**Figure 7:**
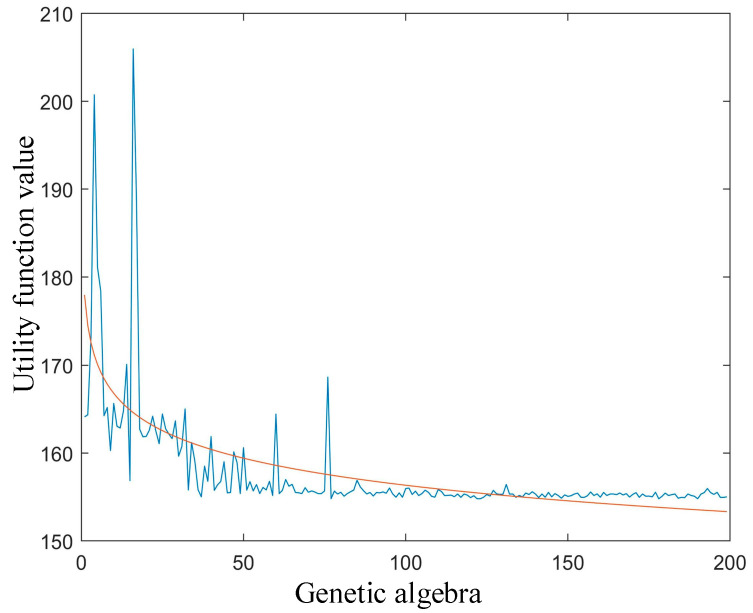
The utility function changes during iteration.

We first examined the convergence of the genetic algorithm in satellite network task deployment. In the simulation experiment, the population size of the genetic algorithm was set as 100, the number of iterations was set as 200, the crossover probability was 0.7, the mutation probability was 0.01, and the generation gap was set as 0.95. For initiated task nodes, the number of nodes used by the algorithm for deployment was set as nine. The task breakdown number was five—that is, the task was deployed on five nodes where cache space and computing resources were available. The mission input data can be obtained from multiple satellites on the same orbital plane as the mission-origination node. Figure 7 shows the algorithm convergence when the genetic algorithm is used to deal with satellite network task deployment under the state of a random network. As the number of iterations continues to increase, the value of the target utility function continues to decrease—that is, the individual’s fitness degree gradually increases and converges after 100 generations of iteration. At the same time, the change data of the utility function are fit to better represent the performance of the algorithm.

**Figure 8:**
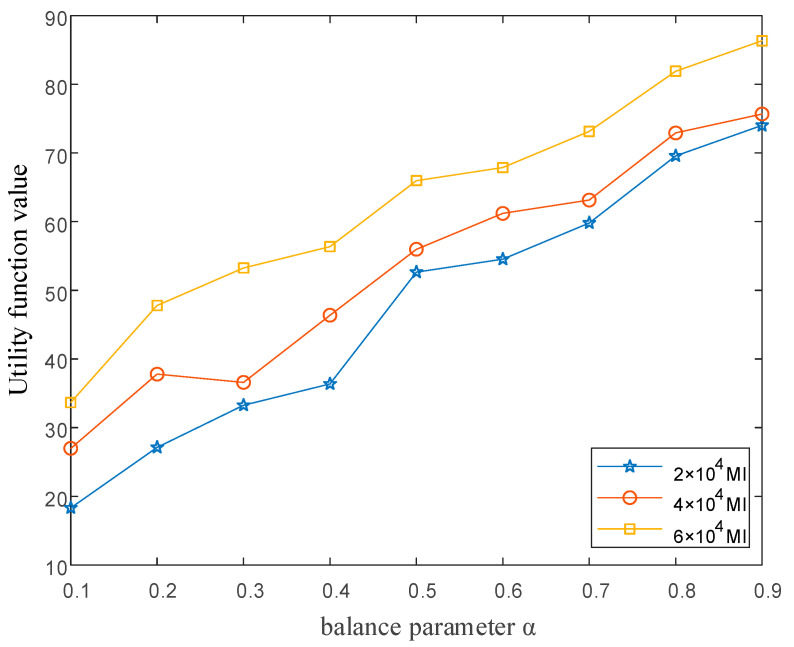
The graph of the value of the utility function as a function of equilibrium parameters.

The computational complexity increases the system response delay. Resource-intensive tasks also bring a large link burden due to the large amount of data required by the tasks. For users, initiated delay-sensitive tasks require the network to do its best to deliver the response. For resource-intensive tasks with time-delay insensitivity, the optimization objective is to reduce the network link burden. We used the method of adding balance parameters to the objective function to balance the relationship between the two. Figure 8 shows the changing relationship between the utility function and the equilibrium parameters. As can be seen, with the increasing of the parameters, the value of the utility function also keeps increasing. The increment parameter indicates the proportion of increment delay in task processing. In addition, we tested the impact of task computation on the utility function, so this experiment added three groups of experiments that kept the data required by the task unchanged. The computation of each task was $2\times 10^{4}$, $4\times 10^{4}$, and $6\times 10^{4}$ MI, respectively. MI here represents the number of machine language execution instructions. From the experiment, it can be seen that after increasing the computation of the task, the value of the utility function increased significantly. The reason for this is that the increase in the number of calculations will deploy the task on the resource available node farther away from the initiating node, which will increase the response delay. The increase in the number of calculations will increase the processing delay consumed by the calculations.

**Figure 9:**
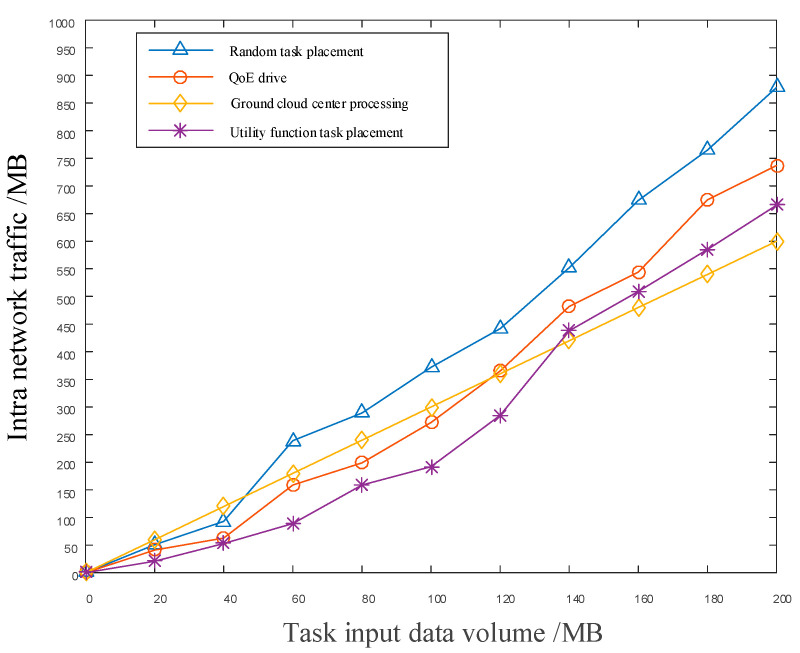
The comparison of network traffic with the amount of task input data.

Figure 9 shows a graph of intranetwork traffic as a function of task input data. In the simulation experiment, the computational amount of the task was set to be unchanged, whereas the amount of data required for task processing was changed. It can be seen that, by increasing the amount of task input data, the network traffic was also increased. The reason for this is that, in the ICN-based communication mode, the communication data are cached in the mission-originating node or in other satellites on the same orbital plane as the mission-originating node. Nevertheless, with further increasing of the task input data, the cache space of the node located closer to the task-initiating node can no longer accommodate the needs of task deployment. Therefore, the task needs to be deployed to the available node farther away, which significantly increases the forwarding times in the network. The utility function task placement proposed in this paper is compared with the strategy of QoE-driven content deployment and random task deployment, as well as sending the task back to the ground cloud computing center. As can be seen in Figure 9, the communication volume brought by the plan of transmitting the mission back to the ground cloud computing center increases linearly. This is because, in using this approach, the data required by the mission can be completely transmitted back to the ground through fixed satellite hops before the calculations can be carried out. The utility function task deployment proposed in this paper has good performance in reducing the traffic caused by the task deployment.

**Figure 10:**
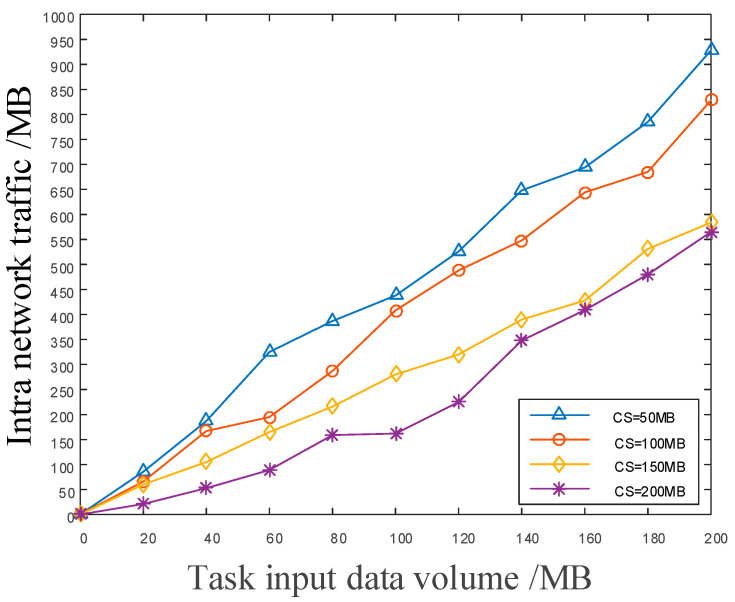
The diagram of the effect of on-board cache capacity on network traffic.

In Figure 10, we continued to investigate the influence of the cache space size of satellite nodes on the intranetwork traffic. In the simulation experiment, the average cache space of the satellite node was set to be within the range of 0–200 MB. It can be seen from the experiment that there is a small gap between the traffic in the network and the input amount of small tasks. The reason for this is that the cache node can be accommodated when the amount of task data are small. However, as the amount of task data increases continuously, the network with larger average cache space can perform better.

**Figure 11:**
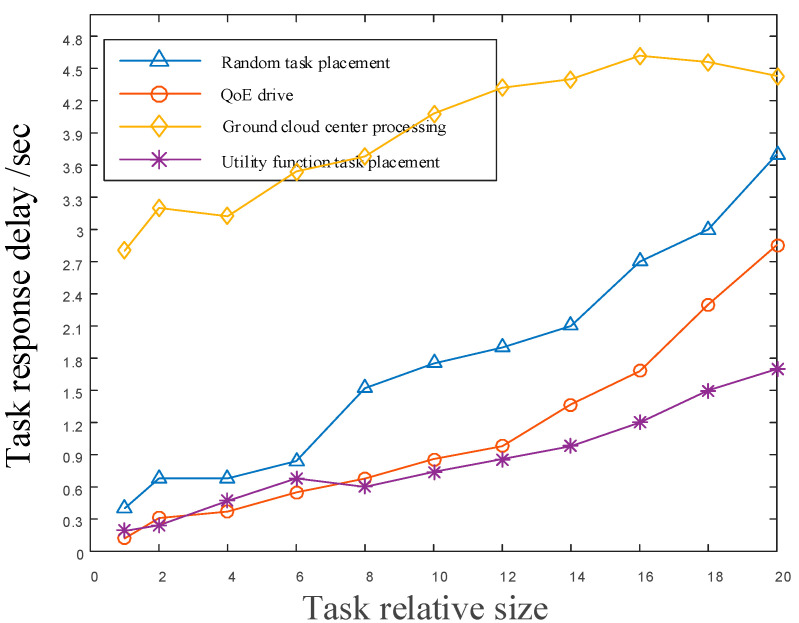
The delay performance comparison of different task placement strategies.

Figure 11 shows the comparison of task processing response delays of different relative sizes under different content placement strategies. As can be seen from the figure, the utility function task placement proposed in this paper can still guarantee a low response delay when the task size keeps getting larger. When the task size is low, the low delay response can be achieved by using the LEO satellite network. When the task scale is relatively low, the data required by the task are transmitted back to the ground computing center and the calculation results are transmitted back to the terminal through the satellite network as the main part of the delay. With the doubling of the size of the mission, the transmission of the data required by the satellite network not only increases the response delay of the mission, but also increases the communication burden of the satellite network. When a task is deployed in a satellite network, due to the limited computing and caching resources of the satellite network, and when the task size is more than 10 times, it will cause a higher task response delay because the task is deployed to a remote resource available node.

## 6. Conclusions

In this paper, the satellite networks based on SDN and ICN were introduced. We then analyzed the application status of edge computing in the satellite networks. Analyzing the scenario of a multilayer satellite network based on the SDN, we modeled the task assignment problem in edge calculation. We then adopted the genetic algorithm to obtain the solutions to the task deployment problem. We also tested the connectivity of the satellite network and the performance of the proposed satellite network deployment method. The simulation results showed that the proposed task deployment algorithm can reduce the processing delay of computing tasks and the network traffic caused by task processing.

## Figures and Tables

**Table 1:** Simulation parameter.

Parameters	Value
Satellite CPU processing speed	$1\times 10^{3}$ MIPS
Ground computing center processing speed	$1\times 10^{8}$ MIPS
Task calculation	[$2\times 10^{4}$, $6\times 10^{4}$] MI
On-board cache space	[20, 100] MB
Number of task decomposition	5
Intersatellite link bandwidth	100 Mbps
Satellite–ground uplink and downlink bandwidth	200 Mbps

## Data Availability

The processed data required to reproduce these findings cannot be shared as the data also forms part of an ongoing study.
